# Potent efficacy of metronomic topotecan and pazopanib combination therapy in preclinical models of primary or late stage metastatic triple-negative breast cancer

**DOI:** 10.18632/oncotarget.6377

**Published:** 2015-11-24

**Authors:** Teresa Di Desidero, Ping Xu, Shan Man, Guido Bocci, Robert S. Kerbel

**Affiliations:** ^1^ Biologic Sciences Platform, Sunnybrook Research Institute, Toronto, Canada; ^2^ Divisione di Farmacologia, Dipartimento di Medicina Clinica e Sperimentale, University of Pisa, Pisa, Italy; ^3^ Department of Medical Biophysics, University of Toronto, Toronto, Canada

**Keywords:** metronomic chemotherapy, topotecan, pazopanib, metastatic triple negative breast cancer

## Abstract

Metronomic chemotherapy has shown promising activity in numerous preclinical studies and also some phase II clinical studies involving various tumor types, and is currently undergoing phase III trial evaluation. Triple-negative breast cancer (TNBC) is an aggressive histological subtype with limited treatment options and very poor prognosis following progression after standard chemotherapeutic regimens. Herein, we evaluated the potential therapeutic impact and molecular mechanisms of topotecan administered in a continuous low-dose metronomic (LDM) manner, alone or in concurrent combination with pazopanib, an antiangiogenic tyrosine kinase inhibitor (TKI), in a triple-negative, primary and metastatic breast cancer orthotopic model; potential molecular mechanisms of efficacy were also studied, especially the impact of hypoxic conditions. The combination of metronomic topotecan and pazopanib significantly enhanced antitumor activity compared to monotherapy with either drug and prolonged survival, even in the advanced metastatic survival setting, with a marked decrease in tumor vascularity, proliferative index, and the induction of apoptosis. Significant changes in tumor angiogenesis, cancer cell proliferation, apoptosis, HIF1α levels, HIF-1 target genes and ABCG2 were found both *in vitro* and in tumor tissue. Notably, the pazopanib and metronomic topotecan combination treatment inhibited expression of *HIF1α* and *ABCG2* genes in cells grown under hypoxic conditions, and this was associated with an increased intracellular concentration of the active form of topotecan. Our results suggest a potential novel therapeutic option for the treatment of metastatic triple-negative breast cancer patients.

## INTRODUCTION

Metronomic chemotherapy refers to the close, regular administration of conventional chemotherapy drugs at low, minimally toxic doses, with no prolonged break periods [1]. Integration with an antiangiogenic agent such as TKIs (e.g. sunitinib and pazopanib) or anti-VEGFR-2 monoclonal antibodies (i.e. DC101) with metronomic chemotherapy can greatly enhance antitumor efficacy, with low toxicity [2]. Metronomic chemotherapy has shown encouraging results in a number of phase II clinical trials, including in metastatic breast cancer [3–6], and is currently undergoing phase III trial evaluation [7–9]. One completed randomized phase III trial, called CAIRO3, evaluating metronomic capecitabine plus bevacizumab as a long-term maintenance therapy in first line metastatic colorectal cancer patients, reported significantly improved progression free survival (PFS) rate, the primary endpoint of the study [10]. Topotecan, a topoisomerase I inhibitor, appears to be highly efficacious both in animal models and in human patients when administered for prolonged periods using metronomic dosing [11–15]. Moreover, metronomic oral topotecan showed an enhanced antitumor activity as a result of combination with concurrent administration of pazopanib, in preclinical models of advanced human ovarian cancer [16, 17], aggressive pediatric solid tumors [18], pediatric sarcomas [19], and renal cell carcinoma [20]. Indeed, a phase I trial of this combination schedule has been conducted in persistent gynecologic tumors, without major toxic effects [21]. Pazopanib, approved in 2009 for the treatment of patients with advanced renal cell carcinoma (RCC) [22], is currently being evaluated alone or in combination with capecitabine in breast cancer patients [23, 24]. Several phase I trials were performed to assess the safety and tolerability of pazopanib in patients with advanced solid tumors [25–27], but the activity of pazopanib alone in metastatic breast cancer has been established only in a single arm open-label multicenter phase II trial, where pazopanib provided disease stability in advanced breast cancer and the authors suggested its use in breast cancer in combination with chemotherapeutic drugs and/or other targeted agents [28]. Few preclinical and clinical data are currently available concerning the impact of topotecan in metastatic breast cancer and its metronomic dosing and scheduling concept has not yet been explored in this indication either alone or in combination with TKIs [29]. Among breast cancer subtypes, “triple-negative” is particularly aggressive with limited treatment options available, and associated with a very poor prognosis [30]. The purpose of our study was to investigate metronomic topotecan alone or in combination with pazopanib in a model of a primary established triple-negative or advanced metastatic breast cancer elucidating possible molecular mechanisms accounting for efficacy of this treatment combination.

## RESULTS

### *In vitro* studies

### Pazopanib, sunitinib or topotecan inhibit endothelial and cancer cell proliferation *in vitro*

*In vitro* pazopanib, sunitinib and topotecan inhibited the cell proliferation of HUVEC, HMVEC-d and of 231/LM2-4 in a time- and concentration-dependent manner (see Supplementary Table 1); the 72-h pazopanib, sunitinib and topotecan standard exposures inhibited the proliferation of 231/LM2-4 with an IC_50_ of 11±0.67 μM, 8±0.73 μM and 24±2.07 nM, respectively, being different from those observed in the 144-h metronomic exposure (2.18±0.53 μM, 2.63±0.33 μM and 3.01±0.58 nM, respectively). Moreover, a greater antiproliferative effect of pazopanib, sunitinib or topotecan, in the metronomic 144-h exposure, on HUVECs was found, as demonstrated by the IC_50_ values (1±0.22 μM, 3±0.95 nM and 1±0.13 nM respectively). The cytotoxic activity on proliferating HMVEC-d was similar to that observed for HUVECs (IC_50_ values are reported in Supplementary Table 1).

### Protracted low-dose treatment with topotecan and TKIs modulates expression of HIF1α in endothelial and cancer cells

**Figure 1:**
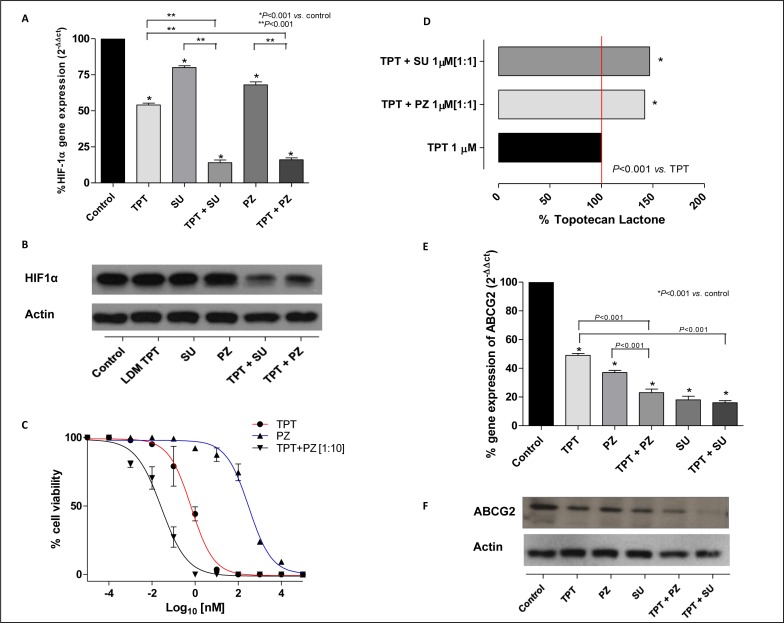
**A.** *HIF1α* gene expression in 231/LM2-4 cells exposed to metronomic topotecan (TPT), pazopanib (PZ), sunitinib (SU) and combinations thereof or with vehicle alone, for 144h in hypoxic conditions. Columns and bars, mean values ±S.E., respectively. *P < 0.001 *vs.* vehicle-treated controls. **B.** HIF1α protein expression in 231/LM2-4 cells exposed to metronomic topotecan (LDM TPT), pazopanib, sunitinib or combinations thereof or with vehicle alone, for 144h in hypoxic conditions. **C.** Sigmoid concentration-effect curve of 231/LM2-4 cells exposed to metronomic TPT, PZ or combinations thereof [1:10], or with vehicle alone for 144h. **D.** Accumulation of topotecan in 231/LM2-4 cell line after exposure to 1 μM topotecan alone and in combination with pazopanib or sunitinib. Columns and bars indicate mean values±S.D., respectively. Small S.D. bars are not visible in the graph. **E.** *ABCG2* gene expression in 231/LM2-4 cells exposed to metronomic topotecan, pazopanib, sunitinib or combinations thereof, or with vehicle alone, for 144 h in hypoxic conditions. Columns and bars, mean values±S.E., respectively. *P < 0.001 *vs.* vehicle-treated controls. **F.** ABCG2 protein level in 231/LM2-4 cells exposed to metronomic topotecan, pazopanib, sunitinib or combinations thereof, or with vehicle alone for 144 h in hypoxic conditions.

After exposure to pazopanib, sunitinib or topotecan and various concurrent combinations of these drugs at concentration corresponding to the experimental IC_50_ of cell proliferation, *HIF1α* gene expression and protein levels were evaluated in cells treated in hypoxic conditions. Low dose topotecan significantly inhibited *HIF1α* gene expression in 231/LM2-4 in hypoxic conditions (54±1.41 % *vs.* 100% of control expression. *P* < 0.001; Figure 1A). As shown in Supplementary Figure 1, a single standard 72h-exposure of topotecan did not decrease hypoxia-induced *HIF1α* gene expression as much as protracted low-dose topotecan, corroborating that the effects of topotecan on *HIF1α* were even more marked when cells were treated with daily administration of low metronomic doses of drug (72h standard exposure 69±0.64% and 144h metronomic daily exposure 54±1.41% *vs.* 100% of control expression. *P* < 0.001; Supplementary Figure 1). In hypoxic conditions both TKIs and low dose topotecan inhibit *HIF1α* gene expression, but the combination significantly reduces this gene expression more than the metronomic topotecan and TKIs alone as follows: pazopanib daily exposure 68±0.14% *vs.* topotecan + pazopanib daily exposure 16±1.37%, and sunitinib daily exposure 80±0.32% *vs.* topotecan + sunitinib daily exposure 14±1.87% if compared to 100% control expression (Figure 1A). Also the HIF1α protein level (Figure 1B) confirmed this marked inhibition when compared to LDM topotecan or TKIs alone.

### Pazopanib or sunitinib enhances the effect of protracted low-dose treatment with topotecan in an hypoxic environment

The combination of topotecan with pazopanib enhances the anti-proliferative effect on 231/LM2-4 cells of the individual drugs used alone, as shown in Figure 1C. To investigate in more detail the basis of this enhanced effect, we measured the intracellular concentration of topotecan lactone in treated cells. Higher topotecan lactone concentrations were found in 231/LM2-4, MDA-MB-231 and endothelial cells exposed to the drugs in combination with either pazopanib or sunitinib, when compared with treatment using topotecan alone. Our results show that at extracellular topotecan concentrations of 1 μM, intracellular accumulation of topotecan is significantly higher (approximately 50%) if the chemotherapeutic drug is co-administered with pazopanib or sunitinib in 231/LM2-4 (Figure 1D; 0.024±0.008 (100%) *vs.* 0.035±0.022 (141%) and 0.036±0.014 (146%) ng/μg protein, respectively; *P* < 0.001), in MDA-MB-231 (Supplementary Figure 2A) and in endothelial cells (Supplementary Figure 2B and 2C).

Based on these findings, we investigated the variation of ABCG2 expression; the gene expression and the protein levels of this transporter were quantified in cancer and endothelial cell lines exposed to the different drugs at the experimental IC_50_ in hypoxic conditions. Similar to HIF1α, Figure 1E shows a significant decrease of *ABCG2* gene expression in 231/LM2-4 hypoxic-treated cells, by the concurrent combination of either TKI and metronomic topotecan (topotecan + pazopanib daily exposure 23±2.57% and topotecan + sunitinib daily exposure 16±1.43% *vs.* 100% of control expression; *P* < 0.001). Moreover, the combination of the two drugs showed a decrease of ABCG2 protein level (Figure 1F) when compared to the single drugs alone, as demonstrated by Western blot analysis.

Moreover, the significantly decreased levels of HIF1α was associated with the decrease of its target genes CA9 (data not shown), VEGF (Supplementary Figure 3) by the treatment combination of metronomic topotecan and pazopanib in 231/LM2-4 and endothelial cells grown under hypoxic conditions.

### The combination of topotecan and pazopanib or sunitinib enhances tumor cell apoptosis

**Figure 2:**
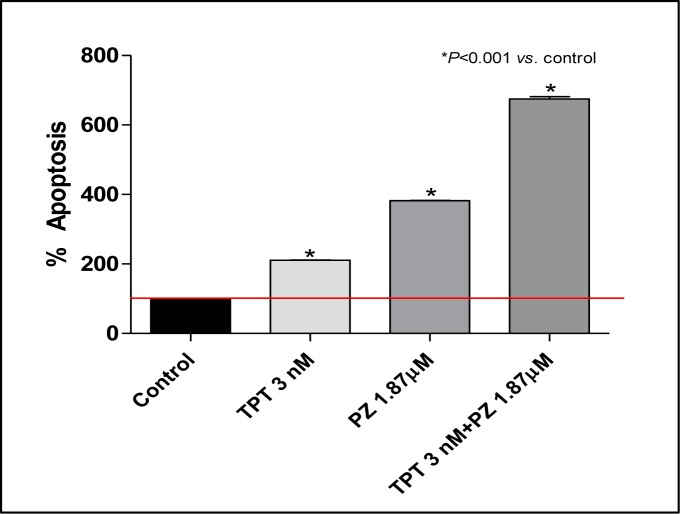
Proapoptotic effects of topotecan (TPT) and pazopanib (PZ) on proliferating 231/LM2-4 cells treated for 144h in hypoxic conditions Apoptosis measurements using the Cell Death Detection ELISA Plus kit (Roche Diagnostics). Percentage Absorbance values are representative of 231/LM2-4 cytosolic nucleosomes. The experiment was repeated 3 times with a least 2 replicates per sample. Columns and bars, Mean values±S.E., respectively. *, *P* < 0.001 *vs.* vehicle-treated controls. Small error bars are not visible in the graph.

The possible impact of the enhanced intracellular topotecan retention caused by pazopanib co-treatment on tumor cell apoptosis was investigated by assessing the mono- and oligonucleosomes in the cytoplasmic fraction of cell lysates and by bromodeoxyuridine (BrdU) incorporation. The extent of DNA fragmentation was increased by the combination of both drugs. In particular, the presence of chromatin fragments was clearly detectable after 144 h for pazopanib and topotecan, alone and in combination at their IC_50_ concentrations. As shown in Figure 2, after 144 h treatment with pazopanib and topotecan (in hypoxic conditions) a significantly higher percentage of apoptotic cells in the treated samples was found when compared to vehicle-treated cells. Furthermore, the same pro-apoptotic effects of pazopanib and topotecan, alone and in combination, were observed also in endothelial cells (HUVECs) at lower concentrations (Supplementary Figure 4). The increase of apoptotic cells was observed also after bromodeoxyuridine (BrdU) incorporation. The addition of pazopanib to metronomic topotecan induced an increase in the percentage of 231/LM2-4 BrdUrd-unlabeled cells, compared to controls (data not shown).

### *In vivo* therapy: effects of single agent topotecan administered as MTD (maximum tolerated dose) or low-dose metronomic schedule (LDM) in combination with pazopanib on localized primary breast tumors

**Figure 3:**
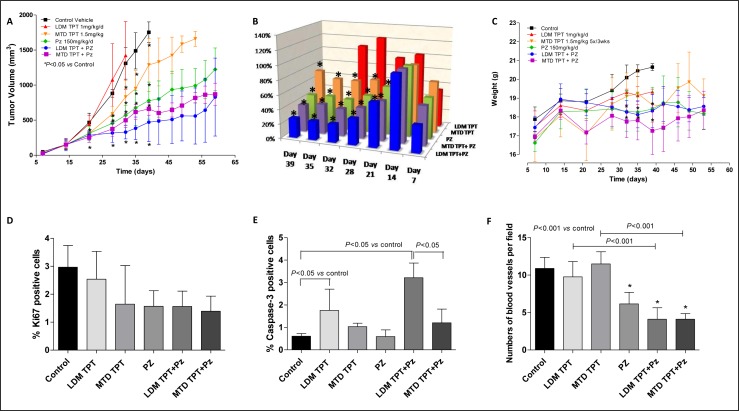
*In vivo* antitumor effects of the single drugs and simultaneous combination of maximum tolerated dose (MTD) or low-dose metronomic (LDM) topotecan (TPT) and pazopanib (PZ) schedules on 231/LM2-4 tumours xenotransplanted in mice: tumor growth curve **A.** % T/C value **B.** and body weights of mice **C.** *P* < 0.05 *vs.* control. Quantification of Ki67 **D.** Capase-3 **E.** and CD31 **F.** in 231/LM2-4 tumour xenografts administered with vehicle, oral topotecan (LDM) at 1 mg/kg daily by gavage, MTD topotecan 1.5 mg/kg 5 days consecutively every 3 weeks through i.p. injection, pazopanib 150 mg/kg by gavage daily without interruption and their combinations. Symbols/columns and bars, mean values±S.D., respectively.

We first tested topotecan and pazopanib on primary orthotopic implanted 231/LM2-4 tumors. We assessed tumor growth in six different treatment groups: vehicle control, LDM topotecan, MTD topotecan, and the doublet combination of LDM/MTD topotecan and pazopanib. As shown in Figure 3A and 3B, single-agent LDM and MTD topotecan did not have any significant antitumor effect. In contrast, the LDM topotecan and pazopanib combination caused a significant tumor growth delay (e.g. at day 35, 384.01 mm^3^ *vs.* 1488.5 mm^3^ of controls, *P* < 0.05), greater than that observed with the MTD combination (619.59 mm^3^). The toxicity profile showed that mice treated with the LDM combination schedule maintained stable body weight throughout the course of the experiment, whereas in the MTD combination treatment group a tumor growth delay was detected in parallel with fluctuating weight loss (e.g. at day 35, 17.82±1.04 g *vs.* 20.46±0.31 g of controls, *P* < 0.05) (Figure 3C). Mice treated with 0.1% HPMC (vehicle control) and 1 mg/kg/day LDM topotecan had to be sacrificed at day 39 and 32, respectively, due to tumor size reaching 1.7 cm^3^.

Staining for Ki67, a marker of cell-cycle progression and proliferation, showed a remarkable decrease in 231/LM2-4 tumors after MTD and LDM combination treatments (Figure 3D). Basal levels of caspase-3 activity detected in tumor samples from control mice were not modified by pazopanib treatment but, conversely, they were increased in the group treated with LDM and MTD topotecan alone. Moreover, the highest significant increase of caspase-3 activity was observed when mice were treated daily with oral topotecan plus concurrent pazopanib (Figure 3E). Thus, the *in vivo* prolonged exposure of tumor cells to MTD/metronomic doublet translates into a significant increase in tumor cell apoptosis. Furthermore, CD31 staining in tumor sections from control, LDM and MTD topotecan treated animals showed a similar presence of number of vessels, which instead were statistically decreased in mice treated with pazopanib alone and in combination with both LDM and MTD topotecan (Figure 3F).

**Figure 4:**
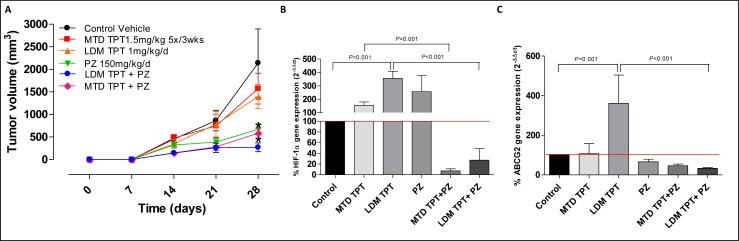
**A.** Tumor growth curve of the single drugs and simultaneous combination of topotecan (TPT) and pazopanib (PZ) schedules in 231/LM2-4 tumour xenografts administered for 14 days with vehicle, oral low-dose metronomic (LDM) topotecan at 1 mg/kg daily by gavage, maximum tolerated dose (MTD) topotecan 1.5 mg/kg 5 days consecutively every 3 weeks through i.p. injection, pazopanib 150 mg/kg by gavage daily without interruption, and their combinations. *P* < 0.05 *vs.* vehicle-treated controls. **B.** and **C.** *HIF1α* and *ABCG2* gene expression in tumor samples, respectively. Columns and bars, mean values±S.E., respectively.

To evaluate the *in vivo* relationship between HIF1α and ABCG2 and their potential roles in the mechanisms involved in the activity of the treatment combination of topotecan with pazopanib, a different experiment on primary tumors was carried out. The 231/LM2-4 variant was injected into MFPs of female SCID mice (*n* = 5 animals per group). When primary tumors reached a size of 150 mm^3^, treatment with topotecan LDM 1 mg/kg/day and MTD 1.5 mg/Kg, pazopanib 150 mg/Kg/day was initiated. After three weeks animals were sacrificed and tumors were excised (Figure 4A). Figure 4B and 4C showed gene expression inhibition of *HIF1α* and *ABCG2*, respectively, in tumors induced by both the MTD and LDM topotecan plus pazopanib concurrent drug combination. The outcomes observed using the two different types of combined treatment protocols were statistically significant when compared with the single administration of topotecan both in MTD and LDM (*P* < 0.001 *vs.* control).

### Survival of mice with advanced metastatic disease

**Figure 5:**
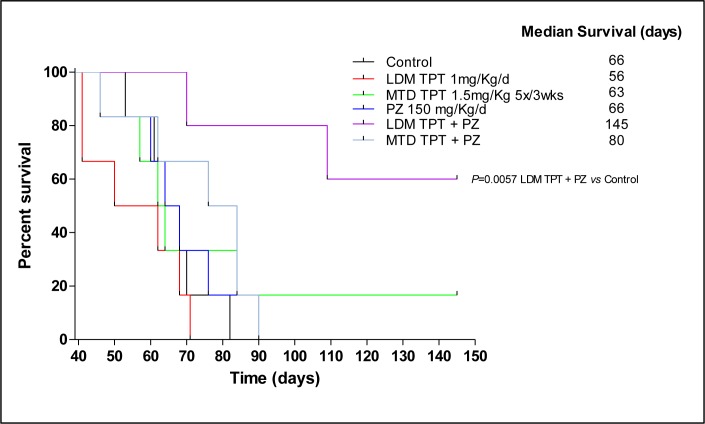
Effect on survival caused by low-dose metronomic (LDM) oral topotecan (TPT) alone or in combination with pazopanib (PZ) The drug treatments and doses tested were: vehicle control, maximum tolerated dose (MTD) topotecan (1.5 mg/kg i.p. 5 consecutive days every 3 wk), LDM oral topotecan (1 mg/kg by gavage, daily), pazopanib once a day (150 mg/kg by gavage daily), pazopanib once a day + LDM topotecan or MTD topotecan. Mice were euthanized when more than 20% body weight loss occurred or when moribund and then assessed; *n* = 5 mice for all groups. Kaplan-Meier *P* < 0.05 was taken as statistical indication of difference *vs*. vehicle-treated controls.

In the experiment involving treatment of advanced metastases the MDA-MB-231/LM2-4 variant was injected orthotopically (*n* = 5 mice per group) and impact on survival was assessed. When tumors reached 400 mm^3^ primary tumors were surgically resected. Treatment was initiated at day 39, nineteen days post primary tumor resection. A Kaplan-Meier survival curve was plotted accordingly for all treated groups is shown in Figure 5. Using survival as the end point, 1 mg/kg/d LDM topotecan therapy alone, pazopanib 150 mg/kg/d monotherapy and MTD topotecan plus 150 mg/kg/d pazopanib group did not have any significant effect on survival of the mice; the MTD topotecan group showed a small effect in prolonging survival. In marked contrast, the chronic metronomic combination treatment (1 mg/kg/d topotecan + 150 mg/kg/d pazopanib) greatly prolonged survival of the mice. Median survival values were as follows: 66 days for control, 56 days for LDM topotecan, 84 days for MTD topotecan, 66 days for Pazopanib, 63 days for MTD topotecan and Pazopanib, and 145 days (*P* = 0.0057 *vs.* control) for LDM topotecan + pazopanib. Notably, our data in the two different models demonstrated that the treatment efficacies in the primary tumours did not necessarily predict for analogous effects in the metastatic setting. Indeed, primary tumor growth reduction was observed (Figure 3A) following LDM or MTD topotecan and pazopanib therapy, but in the advanced metastatic model, only LDM topotecan and pazopanib treated mice had a significant improved overall survival (Figure 5).

### Effects of single agent topotecan administered as MTD versus metronomic compared to combination with sunitinib on growth of localized breast primary tumors

**Figure 6:**
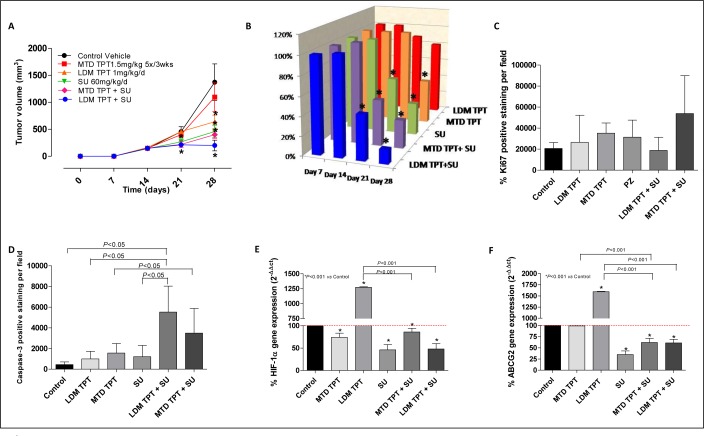
**A.** Effect of low-dose metronomic (LDM) oral topotecan (TPT) alone or in combination with sunitinib (SU). 231/LM2-4 human breast cancer cells were injected i.p. into SCID mice. Treatments were initiated 14 day after tumor injection. The drug treatments and doses tested were: vehicle control, maximum tolerated dose (MTD) topotecan (1.5 mg/kg i.p. 5 consecutive days every 3 wk), LDM oral topotecan (1 mg/kg by gavage, daily), sunitinib once a day (60 mg/kg by gavage daily), sunitinib once a day + LDM topotecan or MTD topotecan. **B.** % T/C value. Quantification of Ki67 **C.** and Capase-3 **D.** levels. Symbols/columns and bars, mean values±S.D., respectively. **E.** and **F.** *HIF1α* and *ABCG2* gene expression in tumor samples, respectively. Columns and bars, mean values±S.E., respectively. *P < 0.001 *vs*. vehicle-treated controls.

In an experiment involving primary tumors in mice treated with sunitinib we assessed tumor growth using six different regimen groups, as described above. As shown in Figure 6A and 6B, single-agent LDM and MTD topotecan did not have any antitumor effect. In contrast, LDM topotecan plus sunitinib caused a greater tumor growth delay (e.g. at day 28, 200.17 mm^3^ *vs.* 1372.6 mm^3^ of controls; *P* < 0.001), greater than that obtained using the MTD drug combination (400 mm^3^). The combination of LDM topotecan and sunitinib showed, although not significant, a slight decrease of Ki67 (+) cells (Figure 6C). Moreover, samples from treated and control mice were analyzed by immunohistochemistry for cleaved caspase-3 expression. Comparisons of stained cells normalized to mm^2^ of tumor area revealed significant increase of cleaved caspase-3 expression in the metronomic combination (Figure 6D; *P* < 0.05). Changes in the gene expression profile of *HIF1α* and *ABCG2* were noted within each group of treated mice. The combination schedule, both LDM and MTD, significantly decreased *HIF1α* and *ABCG2* gene expression (Figure 6E and 6F; *P* < 0.001 *vs.* control and *vs.* the single drug administration, respectively).

## DISCUSSION

Perception of the impact and benefits of antiangiogenic drugs for the treatment of breast cancer remains controversial [31, 32]. On the one hand positive phase III clinical trial results with respect to progression free survival (PFS) have been reported with bevacizumab in combination with various chemotherapy regimens as first line (upfront) therapies in the metastatic setting [31–33] and as a maintenance therapy in a phase III trial where a benefit in overall survival (OS) was also reported in a trial called IMELDA [34]. Neoadjuvant bevacizumab plus chemotherapy also caused increases in pathologic response rates (pCR) in triple negative breast cancer patients [35, 36]. On the other hand the disease free survival results of several adjuvant therapy trials evaluating bevacizumab plus chemotherapy followed by bevacizumab maintenance in early stage breast cancer patients have been disappointing despite a clear initial benefit while patients receive treatment, and for a period after the maintenance treatment is stopped [31, 37]. However, a recent phase III trial of neoadjuvant bevacizumab followed by adjuvant bevacizumab plus chemotherapy (NSABP-B40) reported benefits in PFS and OS in hormone positive-receptor breast cancer patients in secondary analyses [38]. Finally, all phase III trials evaluating an oral antiangiogenic TKI plus standard chemotherapy in metastatic breast cancer patients have been negative [32, 33, 37].

With this background in mind, it would seem that VEGF pathway targeting drugs have anti-tumor activity, albeit limited, against breast cancer in many circumstances, and as such, provides a rationale for devising strategies to improve their impact. Such strategies include reducing toxicity using new treatment combinations (especially incorporating less toxic chemotherapy ‘backbone’ regimens), uncovering promising predictive markers to help select patients more likely to benefit from receiving an antiangiogenic therapy, and developing improved preclinical models to evaluate new treatment possibilities. One such preclinical model involves treatment of mice with advanced metastatic breast cancer, not just primary tumors [39, 40]. As an example, we previously reported that antiangiogenic drugs, including pazopanib or sunitinib were ineffective in treating mice with advanced metastatic human triple-negative breast cancer, even when combined with conventional cytotoxic MTD chemotherapy, whereas the antiangiogenic drugs could cause inhibition of primary tumor growth [41]. However, it has been also described that the antiangiogenic therapy, while blocking tumor volume growth, was found to increase local invasion in multiple primary tumor models, but this effect was blocked by concurrent chemotherapy [42, 43]. These data suggest that the therapeutic impact of antiangiogenic drugs may vary in different tumor models, and that anti-VEGF-A therapy can block the invasion properties of tumor cells in response to chemotherapy [44]. Our metastatic model therapy results recapitulated prior phase III clinical trial outcomes using sunitinib-based regimens to treat metastatic breast cancer patients [41]. We therefore decided to evaluate sunitinib or pazopanib in such metastatic versus conventional primary models in combination with chemotherapy using a low-dose metronomic chemotherapy (topotecan) protocol which has previously shown excellent therapeutic results in other preclinical models including ovarian cancer [16, 17], sarcomas [18] and renal cell carcinoma [20], and investigate mechanisms of action involved in the antitumor activity of this treatment combination.

Triple negative breast cancer has a highly aggressive nature with very limited treatment options and carries a very poor prognosis [30]. A single accepted standard chemotherapy regimen for patients with TNBC is not currently available, and thus treatment is selected from recommended agents approved for metastatic disease (i.e. chemotherapeutic regimens containing anthracyclines, and taxanes alone or in combination with bevacizumab) [30, 45]. Therefore new and more effective therapies for patients affected by this breast cancer subtype are urgently needed [30]. The impact of pazopanib in the treatment of breast cancer will be better known in the near future because of ongoing trials which involve the combination of this TKI with various chemotherapy regimens [23, 24]. However, one important issue concerning such combination treatments will likely be the tolerability of administering a TKI such as pazopanib with standard-of-care cytotoxic chemotherapy agents. Although pazopanib at 800 mg was shown to be safely combined with weekly paclitaxel at 80 mg/m^2^, other chemotherapy regimens resulted in dose reductions of pazopanib [46]. In this regard, low dose, less toxic metronomic chemotherapy, which has shown efficacy in patients with metastatic breast cancer in limited phase II clinical studies [47], could be a feasible combination approach to limit the toxicity of pazopanib or other TKIs and enhance efficacy.

In our study we report, for the first time to our knowledge, the remarkable efficacy of the metronomic topotecan and pazopanib treatment combination, especially in the metastatic triple negative breast cancer model, which caused a significantly prolonged survival times. Although a possible limitation of our study concerns the employment of a metastatic model using a single cell line (i.e. LM2-4), the choice of this metastatic variant subline derived from the human triple negative breast cancer cell line MDA-MB-231, was based on its capability to give rise to extensive systemic spontaneous metastases in multiple sites such as the lungs, liver and lymph nodes within one month after resection of the primary orthotopic tumour in SCID mice [40]. As previously reported, responses of primary tumours are not necessarily indicative of the effects of the same treatment strategies when use to treat metastatic disease [41, 48]. Indeed, our results showed that both the LDM or MTD combinations of topotecan and pazopanib improve primary tumour response compared to the single drugs alone. However, only the metronomic combination was highly efficacious against metastatic disease, highlighting the potential benefit of using metronomic chemotherapy in combination with an antiangiogenic drug during late stage disease settings.

What are the possible underlying mechanisms responsible for these encouraging effects? Topotecan when administered in a metronomic-like fashion was previously reported to potently inhibit HIF1α translation by a DNA damage-independent mechanism [49]. When administered in a low-dose, continuous schedule it blocks the expression of tumor cell HIF1α, as first described by Melillo's group [50]. HIF1α has a central role in regulating a broad spectrum of genes involved in tumor angiogenesis, invasion, and metastasis [51, 52]. Thus, exposure of tumor cells to metronomic topotecan causes a decreased expression of VEGF, likely as a result of HIF1α downregulation [5]. As previously reported, the addition of a low dose daily topotecan protocol with bevacizumab significantly inhibited tumor growth in glioblastoma xenografts, when compared to mice treated with topotecan or bevacizumab alone [49]. In our *in vitro* experiments we showed the ability of low-dose metronomic oral topotecan alone to suppress HIF1α in cells under hypoxic conditions. However, in our breast cancer model no significant antitumor effect on primary tumors was observed and neither prolonged survival was noted. Indeed, even though LDM topotecan could have its therapeutic effects increased in a hypoxic-stressed tumor microenvironment, in our study, the administration of this drug alone in either primary or metastatic breast cancer therapy models did not translate into a therapeutic advantage. One possible explanation for these findings may be the level of ABCG2 expression, a known and effective resistance mechanism of mammary tumors enabling them to evade topotecan-induced DNA damage [53]. Indeed, in our study the metronomic topotecan treatment regimen decreased ABCG2 expression, as shown in Figure 2, but the level of this inhibition was likely not enough to neutralize the effects of the transporter on the drug *in vivo*, and thus lowering the intracellular concentrations of topotecan.

Studies on ABCG2 transporter have revealed three hypoxia-response elements in the ABCG2 promoter, suggesting that its expression is likely regulated by the hypoxia - HIF1α pathway -, that results in modulation of its expression [54], enhancing cell survival [55]. Considering the possibility that HIF1α activation may strongly influence ABCG2 upregulation and that the level of HIF1α expression is not the same in all tumors, we evaluated whether sunitinib or pazopanib could enhance the action of an HIF1α inhibitor such as topotecan. The results of our experiments showed the significantly decreased levels of HIF1α and its target genes - CA9 (data not shown), VEGF and ABCG2 - by the treatment combination of metronomic topotecan and pazopanib in 231/LM2-4 or endothelial cells grown under hypoxic conditions. Moreover, this treatment protocol caused significant tumor growth delays and greatly prolonged survival times of the mice together with a significant decreased *HIF1α*/*ABCG2* gene expression in tumors, when compared with the single drug treatments, suggesting the use of the combination schedule treatment. Similar changes in the expression of HIF1α and ABCG2 were also noted in 231/LM2-4 hypoxic-treated cells and tumors treated with topotecan and sunitinib. Simultaneous treatment of these drugs markedly reduced expression of these genes relative to either agent alone, explaining the high degree of efficacy of the combination compared to the weak or no-effects of LDM topotecan alone.

Interestingly, various TKIs have also been described as competitive inhibitors of ABCG2 [56]. Imatinib mesylate - a substrate for ABCG2 - can reverse resistance to irinotecan acting as such a competitive ABCG2 inhibitor [57]. Our group (T.D.D. and G. B.) previously suggested that the combination of axitinib and SN38, the active metabolite of irinotecan, increased intracellular accumulation of SN38, by axitinib-mediated inhibition of ABCG2 [58]. Sunitinib was found to inhibit transport mediated by ABC drug transporters, which may affect the bioavailability of drugs which are co-administered [56]. Pazopanib, instead, seems to be a high affinity substrate for this carrier, as previously described [25]. Pazopanib could itself be a substrate of ABCG2 and thus mediates a pharmacodynamic interaction with LDM topotecan, resulting in a competitive inhibitory mechanism at the level of topotecan extrusion from the cancer cell mediated by ABCG2. Thus, the results of our study suggest that combining low dose topotecan with TKIs that are substrates of common ABC transporters might downregulate, saturate or inhibit the transporter and increase anticancer activity. Indeed, our *in vitro* data clearly show that the combination of low dose topotecan with pazopanib significantly increase intracellular levels of active topotecan in cancer cells, enhancing the antitumor effects of the combination [20].

Since ABC transporters are also involved in the disposition of most drugs, pharmacokinetic interactions may considerably complicate successful application of combination therapies in the clinic [53]. The pharmacokinetic and pharmacodynamic changes that can occur when chemotherapeutic drugs at standard doses are combined with TKIs may allow to exploit the aforementioned advantage of LDM topotecan therapy in a hypoxic-stressed tumor microenvironment. In this regard, our data showed no changes in mouse body weight associated with metronomic topotecan combination regimens, in contrast with the MTD-based regimens. Moreover, in a clinical study in patients with gynecological cancers treated with this combination drug schedule, no dose adjustment was necessary for low dose topotecan, suggesting a good toxicity profile of the combination of metronomic topotecan and pazopanib [21].

Although at first glance it could appear that there is only a small difference in topotecan dose between the two administered schedules (LDM 1.0 mg/kg p.o. daily *vs.* MTD 1.5 mg/kg i.p. for 5 days every 3 weeks), the route of administration is fundamental to understand the different active plasma concentrations reached by the LDM and MTD protocols. Indeed, as previously demonstrated in a topotecan pharmacokinetic study, after oral administration the maximal plasma concentration (C_max_) of the active lactone was 10-fold less than that reached after i.p. drug administration [59]. Therefore the oral administration and the dose fractionation of the metronomic protocol determines low concentrations of the active drug, and thus an improved toxicity profile, suggesting its possible use for future clinical trials.

Another interesting finding of our study is that pazopanib, sunitinib and topotecan all cause a direct cytotoxic effect on cancer and endothelial cells, as shown by the combined IC_50_. *In vivo*, cancer cell proliferation has been measured by immunohistochemical analyses of Ki67, a nuclear marker expressed in all phases of the cell cycle except G0 [60]. The expression of Ki-67 is one of the most reliable indicators of the proliferative status of cancer cells and is closely associated with the growth and invasion of breast cancer [61]. In our experiments the number of Ki-67-positive cells in tumors obtained from LDM topotecan plus pazopanib-treated mice was 48% lower than found in the control group. Although these data were obtained from tumor tissue samples taken at different time points, in our *in vivo* study on primary tumors - treated with topotecan and pazopanib (Figure 3) - the Ki67 staining was performed approximately two weeks after the beginning of the second cycle of MTD administration. Rapid tumor cell repopulation can take place between successive MTD chemotherapy treatments, but in this experiment Ki67+ cells were not increased in MTD chemotherapy-treated tumors, suggesting that probably tumor regrowth/repopulation during the chemotherapy break period might be more evident after 3 weeks from the beginning of the MTD therapy.

Moreover, in our *in vitro* experiments, we were able to show that pazopanib triggers apoptosis in 231/LM2-4 cells, which was much more pronounced by this orally available drug under hypoxic conditions. In agreement with these findings, low-dose combination treatments were able to significantly increase the activity of caspase-3 expression compared to controls, as noted in *in vivo* models. Caspase-3-like protease is involved in the apoptotic death of MDA-MB-231 and 231/LM2-4 cells [62]. The combinations of LDM topotecan and pazopanib were associated with marked and significantly higher rates of cancer cell apoptosis and resulted in significant decreased levels of vessel density, assessed by CD31 staining.

In conclusion, the combination pazopanib/metronomic topotecan regimen was particularly effective in the metastatic triple-negative breast cancer model including causing a significanting prolonged survival with a favourable toxicity profile. Also noted were marked decreases in tumor volume, vascularity and proliferative index, associated with the downregulation of HIF1α and ABCG2, and increased intracellular concentrations of active topotecan. Furthermore, these changes were highly dependent on growth of the cells under hypoxic conditions when assessed *in vitro*. Also noteworthy were the differences in therapeutic outcomes in the various mice treatment groups when therapy was undertaken in mice with primary tumors *versus* advanced metastatic disease, as also previously discussed by Francia and colleagues [39]. These findings suggest the possibility of the use of low dose metronomic topotecan in combination with pazopanib, or similar drug combinations involving a topoisomerase-I poison with an antiangiogenic drug (e.g. antibodies such as bevacizumab or ramucirumab), as novel and potential therapeutic options for the treatment of triple-negative metastatic breast cancer.

## MATERIALS AND METHODS

### Materials, drugs and cell lines

The details of materials, drugs and cell lines used in the experiments are reported in the Supplementary Materials and Methods.

### *In vitro* studies

*In vitro* assessment of cell proliferation inhibition by topotecan, pazopanib and their combination on triple negative breast cancer cell line MDA-MB 231 cell line [63, 64] and its highly metastatic variant called MDA-MB 231/LM2-4 [40] and on endothelial cells for 72h [65] and 144h [58] was performed by CellTiter 96® AQueous One Solution Cell Proliferation Assay (MTS; Promega) [66]; apoptosis measurements were made by Cell Death Detection ELISA Plus kit (Roche Diagnostics, Laval, QC, Canada) and bromodeoxyuridine (BrdU) incorporation analysis; HIF1α and ABCG2 gene expression was obtained with Real-time PCR analysis and their protein levels evaluation was performed by Western Blot assay; intracellular accumulation of topotecan was measured by HPLC analysis as previously described [67, 68]. The details of these experiments are reported in the Supplementary Materials and Methods.

### *In vivo* studies

### Animals and treatments

Six-week-old in house bred female YFP SCID mice were housed in microisolator cages on vented racks and manipulated using aseptic techniques. MDA-MB 231/LM2-4 is a variant cell line of MDA-MB 231 selected *in vivo* for aggressive spontaneous metastatic spread from established but resected primary tumors and was grown in cell culture as previously described [40]. Mammary fat pad (MFP) orthotopic injection (2×10^6^ cells) was carried out as previously described [40]. Weekly caliper measurements were carried out to determine tumor growth and tumor volume. Tumor volume (mm^3^) was defined as follows: [(w1 x w1 x w2) x (π/6)], where w1 and w2 were the smallest and the largest tumor diameter (mm), respectively. Treatment of primary tumors (n = 5 mice per group) was initiated when the average volume was in the range of 100 - 150 mm^3^. All mice were randomized just before initiation of treatment. In the survival experiments (n = 5 mice per group), surgical resection of the primary tumors was carried out when the average tumor size was around 400 mm^3^. Control mice received either vehicle and/or normal saline as appropriate. Pazopanib, sunitinib and topotecan or their simultaneous combinations using conventional and metronomic schedules were administered as follows: LDM topotecan LDM was given at 1 mg/kg daily by gavage [20]; MTD topotecan was given at 1.5 mg/kg 5 days consecutively every 3 weeks via i.p. injection [16]; pazopanib 150 mg/kg was administered by gavage daily without interruption; sunitinib was administered by gavage at 60 mg/kg dose daily. Each experiment employed the minimum number of mice needed to obtain statistically meaningful results. At termination tumors were excised, measured and sampled for gene expression and immunohistochemistry. Drug efficacy was based on percentage of the average treated-tumour-volume divided by the average vehicle-control-tumour-volume (% T/C) [69].

### Immunohistochemistry

Tumor tissue samples in the primary tumor experiments with topotecan and pazopanib were taken at different time points, when tumors in mice of each group reached their respective volume endpoints. The end of the experiment for each group was as follows: i) control group, day 39; ii) LDM topotecan, day 32; iii) MTD topotecan, day 53; iv) pazopanib, day 59; v) LDM topotecan + pazopanib, day 59; vi) MTD topotecan + pazopanib, day 59. Instead, in the case of topotecan and sunitinib, all samples were taken at day 28, at the end of the experiment. Briefly, tumor tissue samples from all the different treatment groups were fixed in 10% neutral-buffered formalin for 24 h, processed and embedded in paraffin for histology and immunohistochemistry. Formalin-fixed and paraffin-embedded samples were firstly cut into 5-μm-thick sections. The antigen retrieval was accomplished by deparaffinization, rehydration, and heating in a microwavable pressure cooker with citrated buffer; 3% hydrogen peroxide diluted in water was used to block the endogenous peroxidase activity and BSA was used to block nonspecific staining. Sections were incubated with rabbit PECAM-1 antibody (M-20) (1:200, #sc1506, Santa Cruz Biotechnology), rabbit anti-Ki67 antibody (1:1000, #VPK451, VECTOR Lab) and rabbit anti-cleaved Caspase-3 (Asp175) (1:300, #9664, Cell Signaling) at 4°C overnight. Negative controls were carried out by omitting the primary antibodies. The LSAB + System HRP Kit (DAKO, Mississauga, ON, Canada) was used to detect primary antibody followed by staining with DAB reagent and counterstaining with hematoxylin. Sections were visualized under a Carl Zeiss Axioplan 2 microscope (Carl Zeiss Canada Inc) and images captured with a Zeiss Axiocam digital camera controlled by AxioVision 3.0 software.

### Real-time PCR and western blot analysis of HIF1α and ABCG2 on tumor tissue samples

Tumor tissues (from primary tumors) were homogenized using the gentleMACS™ Dissociator (Miltenyi Biotec, San Diego, CA, USA). Total RNA was extracted from tumor tissue samples by using the RNEasy kit mini (Qiagen, Mississauga, ON, Canada) following manufacturer's instructions. Human HIF1α and ABCG2 gene expression and protein levels evaluation in tumor samples was performed as described in Supplemental materials and methods.

### Statistical analysis

The results of all *in vitro* experiments were subjected to analysis of variance among groups (ANOVA), followed by the Student-Newman-Keuls test. Tumor therapy results are reported as mean±S.D. Survival curves were plotted by the method of Kaplan and Meier and were tested for survival differences using the log-rank test. The level of significance was set at *P* < 0.05. Statistical analyses were performed using the GraphPad Prism software package version 5.0 (GraphPad Software, Inc, San Diego, CA).

## SUPPLEMENTARY MATERIALS AND METHODS, TABLE AND FIGURES


