# Supplementary material for: Potent efficacy of metronomic topotecan and pazopanib combination therapy in preclinical models of primary or late stage metastatic triple-negative breast cancer
Source: Oncotarget. 2015 Nov 24;6(40):42396–410. doi: 10.18632/oncotarget.6377 (PMC4767441; doi:10.18632/oncotarget.6377)
Supplement: Supplementary file 1 [file oncotarget-06-42396-s001.pdf]

Potent efficacy of metronomic topotecan and pazopanib combination therapy in preclinical models of primary or late stage metastatic triple-negative breast cancer

Supplementary Materials and Methods

Materials and drugs

Recombinant human vascular endothelial growth factor (rhVEGF) and recombinant human epidermal growth factor (rhEGF) were purchased from R&D Systems Inc. (Minneapolis, MN, USA). MCDB131 medium, type A gelatin from porcine skin, supplements and all other chemicals not listed in this section were purchased from Sigma-Aldrich (Oakville, ON, Canada); RPMI 1640 medium (Hyclone) and fetal bovine serum (FBS) (Hyclone, Logan, Utah) were purchased from Fisher Scientific (Markham, ON, Canada); horse serum (HS), L-glutamine, penicillin, streptomycin, antibiotics were from Life Technologies (Burlington, ON, Canada). Tissue culture plasticware (Nunc, Thermo Scientific) were supplied by VWR (Mississauga, ON, Canada). Topotecan, pazopanib, and the pazopanib vehicle, hydroxypropylmethyl cellulose, were supplied by GlaxoSmithKline. Sunitinib was purchased from LC Laboratories, Woburn, MA, USA. For the *in vitro* experiments the drugs were dissolved in DMSO and diluted as needed; for *in vivo* studies these drugs were reconstituted as per the manufacturer's instructions.

Cell lines

Human Umbilical Vein Endothelial Cells (HUVECs) and Human Dermal Microvascular Endothelial Cells (HMVEC-d) were purchased from Clonetics (San Diego, CA) and maintained in MCDB131 medium supplemented with antibiotics, 10% heat-inactivated FBS, L-glutamine (2 mM), heparin (10 IU/ml), rhEGF(10 ng/ml) and bFGF (5 ng/ml). The human triple-negative breast cancer cell line MDA-MB-231 [64] was obtained from Jeff Lemontt (Genzyme) and maintained in RPMI1640, supplemented with 5% FBS. A highly metastatic variant of MDA-MB-231, called 231/LM2-4, isolated in vivo as previously described [40] was cultured in RPMI 1640

supplemented with 5% fetal bovine serum. All the *in vitro* experiments, for the exceptions of cell proliferation assays, were performed in hypoxic (1% (v/v) O_2, 5% (v/v) CO_2, 95% humidity) conditions. Cells were cultured under normoxic conditions (20% O_2, 5% CO_2, 95% humidity) until 70%–80% confluent, and were then switched to hypoxic conditions (1% O_2, 5% CO_2, 95% humidity) in an Invivo2 hypoxia chamber (Ruskinn Technologies).

Animals

In house bred female YFP severe combined immunodeficient (SCID) mice were used for all *in vivo* experiments. The Sunnybrook Research Institute Animal Care Committee (SRI ACC) has approved the *in vivo* study. The procedures involving animals and their care were conducted in strict conformity with the animal care guidelines of Sunnybrook Research Institute and the Canadian Council of Animal Care (CCAC).

Cell line authentication

Cell line authentication was carried out by genotyping, and the cells were confirmed to be human in origin. All human cell lines used in this publication were processed by Genetica DNA Laboratories (a LabCorp Specialty Testing Group; Burlington, NC) for authentication testing using analytical procedures for DNA extraction, PCR and capillary electrophoresis on a 3130xl genetic analyzer (Applied Biosystems). The thirteen core CODIS short tandem repeat (STR) loci plus PENTA E and PENTA D, and the gender-determining locus, amelogenin, were analyzed using the commercially available PowerPlex® 16HS amplification kit (Promega Corporation) and GeneMapper ID v3.2.1 software (Applied Biosystems). Appropriate positive and negative controls were used concurrently throughout the analysis. Authentication of each cell line was confirmed by entering the STR DNA profile of each tested cell line into known repository cell line databases [i.e. ATCC, DSMZ, etc]; authentication is defined as having a percent match with the reference STR profile at or above 80% when using the ANSI/ATCC guidelines (ASN-0002-2011) OR having a “unique” STR DNA profile (no matches found) for “in-house” cell lines not

distributed by any cell line repository. Routine mycoplasma screening was carried out in-house using commercial kit (MycoAlert Detection Kit, Lonza, Mississauga, ON, Canada), which confirmed the cell line was mycoplasma free.

Cell proliferation assay

HUVECs, HMVEC-d (1 x 10^3 cells/well) and 231/LM2-4 (1 x 10.5^3 cells/well) cells were plated in 24-well plates (1% gelatin-coated for HUVEC and HMVEC-d) and allowed to attach overnight. Cells were treated with pazopanib (0.01-1000 nM), sunitinib (0.01-1000 nM) and topotecan (0.001-1000 nM), alone or in combination, or with their respective vehicles continuously for 144 h in 0.1 ml of medium, adding fresh medium and drug every 24 h to mimic continuous metronomic treatment [61]. In separate experiments, cells were treated with pazopanib (0.01-1000 nM), sunitinib (0.01-1000 nM) and topotecan (0.001-1000 nM), alone or in combination, or with their respective vehicles for 72 h to mimic maximum tolerated dose (MTD) treatment [58]. At the end of treatments, the cells were harvested with trypsin/EDTA, and the viable ones counted with a hemocytometer. Cell viability was assessed by trypan blue dye exclusion. The data are presented as the percentage of the vehicle-treated cells [58]. An additional method for determining the number of viable cells undergoing proliferation was performed. Endothelial and tumor cell lines were plated in 96-well plates (6 x 10^2 cells) and treated, as previously described, for 72h or continuously for 144 h [65]. Proliferation assays were performed using CellTiter 96® AQueous One Solution Cell Proliferation Assay (MTS; Promega) according to manufacturer's instructions [66]. All the experiments described were performed both in normoxic conditions. Values were expressed relative to vehicle-treated cells. The concentration of drugs that decreased cell proliferation by 50% (IC_{50}) as compared to controls was calculated by non-linear fitting of experimental data. All experiments were repeated, independently, three times with at least 9 samples for each concentration.

Apoptosis measurements and Bromodeoxyuridine Incorporation Assay

To quantify the apoptotic process, cancer cells were treated for 144 h with topotecan and pazopanib at a concentration corresponding to the experimental IC_{50} of cell proliferation, alone and in simultaneous combination or with their vehicles in hypoxic conditions. At the end of the experiment, cells were collected and the Cell Death Detection ELISA Plus kit (Roche Diagnostics, Laval, QC, Canada) was used. All the absorbance values were plotted as a percentage of apoptosis relative to control cells (vehicle only), which will be labeled as 100%. All experiments were repeated three times with at least three replicates per sample. For bromodeoxyuridine (BrdU) incorporation analysis, 24 h after seeding, LM2-4 cells were treated with the simultaneous combination of topotecan and pazopanib at their experimental IC_{50} and pulsed with BrdU for 18 h (BD Bioscience). Cells were fixed and stained using a FITC BrdU Flow Kit in accordance with the manufacturer's instructions (BD Biosciences). Populations in G0-G1, S, G2-M phase and apoptotic cells were measured by flow cytometry with a FACScalibur (BD Biosciences). A total of 20,000 events were counted for each sample. Data were analyzed with FlowJo software (Tree Star). In each instance, flow cytometry was performed at least twice.

Real-time PCR analysis of *VEGF*, *HIF1α* and *ABCG2* gene expression on tumor and endothelial cells

To evaluate the expression of the genes encoding human *VEGF*, *HIF1α* and *ABCG2* proteins, $6x10^4$ HUVEC, $2x10^4$ 231/LM2-4 cells were grown in their respective media and treated with pazopanib, sunitinib, topotecan or concurrently in combination at a concentration corresponding to the experimental IC_{50} of cell proliferation or with vehicle alone, continuously for 144 h in hypoxic conditions. Total RNA was extracted from samples by using the RNEasy kit mini (Qiagen). Briefly, RNA (1 μg) was reverse transcribed using Omniscript RT Kit (Qiagen, Germantown, MD), according to the manufacturer's protocol. The resulting cDNA was diluted

(2:3) and then amplified by SYBR green-based real-time RT-PCR (QuantiTect® SYBR Green PCR Kit, QIAGEN) was performed using an ABI Prism 7000 Sequence Detection System (Applied Biosystems) with gene-specific primers purchased from Applied Biosystems. The polymerase chain reaction (PCR) thermal cycling conditions and optimization of primer concentrations were followed as per manufacturer's instructions. Amplifications were normalised to HMBS (Hs_HMBS_1_SG, QuantiTect® Primer Assay Qiagen), and the quantitation of gene expression was performed using the ΔΔCt calculation, where Ct is the threshold cycle; the amount of target, normalised to the endogenous control and relative to the calibrator (vehicle-treated control cells), is given as $2^{-\Delta\Delta Ct}$. All experiments were repeated, independently, three times with at least 9 samples for each concentration.

High Performance Liquid Chromatography (HPLC) analysis of topotecan concentrations in endothelial and cancer cells

HUVEC, HMVEC-d, MDAMB231 and 231/LM2-4 cells (1×10^6) were incubated in RPMI 1640 supplemented with 5 % FBS, in 10 cm^2 dishes for 16 h at 37 ˚C. Topotecan stock solution in DMSO was diluted in pH 3 PBS (37 ˚C) to convert the compound into their active lactone. Topotecan [1 µM] was diluted in RPMI medium just before the start of the incubation. Cells were treated with topotecan [1 µM], pazopanib [1 µM], sunitinib [1 µM] or their concurrent combinations at a ratio of [1:1]. After incubation for 5 min at 37 ˚C, the treatment medium was removed; cells were washed twice with cold PBS at pH 5 and scraped immediately. The cells were then ultrasonically lysed (10 s x 3). The lysate (200 µL) was added to 400 µL cold methanol and centrifuged (5 min, 7,500 g, 4 ˚C) for topotecan extraction to determine the intracellular drug concentration. Proteins in the remaining lysate were quantified by colorimetric assay (Bio-Rad Laboratories) [67]. The concentration of topotecan in the sample

was measured using a previously described sensitive high-performance liquid chromatography (HPLC) method [68].

Western Blot analysis

231/LM2-4 breast cancer cells grown in RPMI 1640 supplemented with 5 % FBS were treated with topotecan, pazopanib and sunitinib at a concentration corresponding to the experimental IC_{50} of cell proliferation or with their simultaneous combination in hypoxic conditions. At the end of the experiment, cells were lysed in protein lysis buffer (Cell Signaling Technology, Danvers, Mass) supplemented with 1 mM phenylmethanesulfonyl fluoride, 2 µg/mL aprotinin, and 1% phosphatase inhibitor cocktail, then stored at −80°C. Proteins (20 µg) were loaded equally onto 10% polyacrylamide gels, and electrophoresis was performed under denaturing conditions. Proteins were transferred onto polyvinylidene difluoride membranes, which were blocked in a 5% milk TBS-T solution for 1 hour then incubated overnight with each of the following primary antibodies: Anti-HIF1α antibody (diluted 1:500, Abcam), Anti-BCRP/ABCG2 antibody [BXP-21] (1:1000, Abcam), and mouse monoclonal anti-human b-actin antibody (1:5000; Sigma-Aldrich). Membranes were incubated with either goat anti-mouse horseradish peroxidase (HRP) (1:5000, Sigma-Aldrich) for 30 minutes, followed by chemiluminescence detection solutions (ECL Plus; G.E. Healthcare, Baie d'Urfe, QC, Canada) and exposure to x-ray film (Kodak, Rochester, NY). Densitometric analysis was performed by ImageJ software (National Institutes of Health, Bethesda, Maryland); loading was normalized using bands for β-actin.

References are numbered as in the reference section of the manuscript

Supplementary table 1. IC_{50} values of topotecan, pazopanib and sunitinib on 231/LM2-4, HUVEC and HMVEC-d cells treated for 72h and 144 h. Results are expressed as mean ± S.D. Standard, standard *in vitro* treatments.

**LM2-4**	**Standard 72h**	**Metronomic 144h**
Topotecan	24±2.07 nM	3.01±0.58 nM
Pazopanib	11±0.67 µM	2.18±0.53 µM
Sunitinib	8±0.73 µM	2.635±0.33 µM
**HUVEC**		
Topotecan	17.68±3.13 nM	1±0.13 nM
Pazopanib	1.74±0.45 µM	1±0.22 µM
Sunitinib	11±0.17 nM	3±0.95 nM
**HMVEC-d**		
Topotecan	15.04±2.9 nM	1±1.26 nM
Pazopanib	2.46±3.4 µM	0.45±0.047 µM
Sunitinib	12.97±7.69 nM	4±3.78 nM

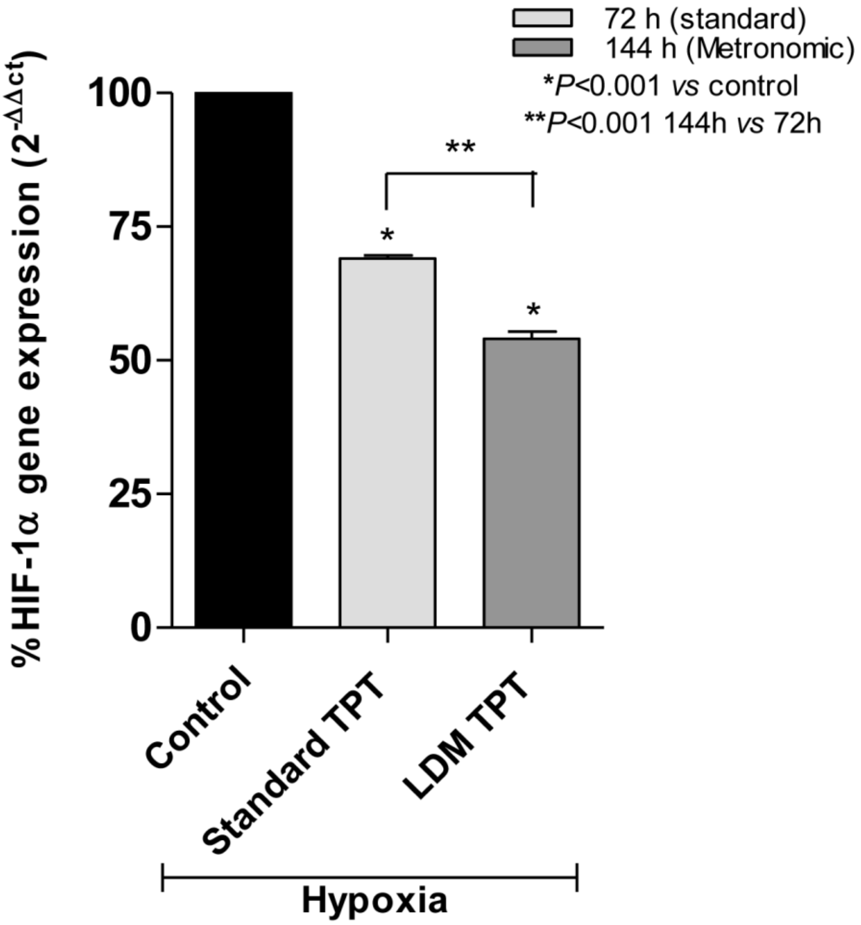

Supplementary figure 1. *HIF1α* gene expression in 231/LM2-4 cells exposed to topotecan (TPT) 24 nM (standard *in vitro* treatment; Standard TPT), 3 nM (low dose metronomic; LDM) or with vehicle alone for 72 h or 144 h in hypoxic conditions. Columns and bars, mean values ± S.E., respectively.

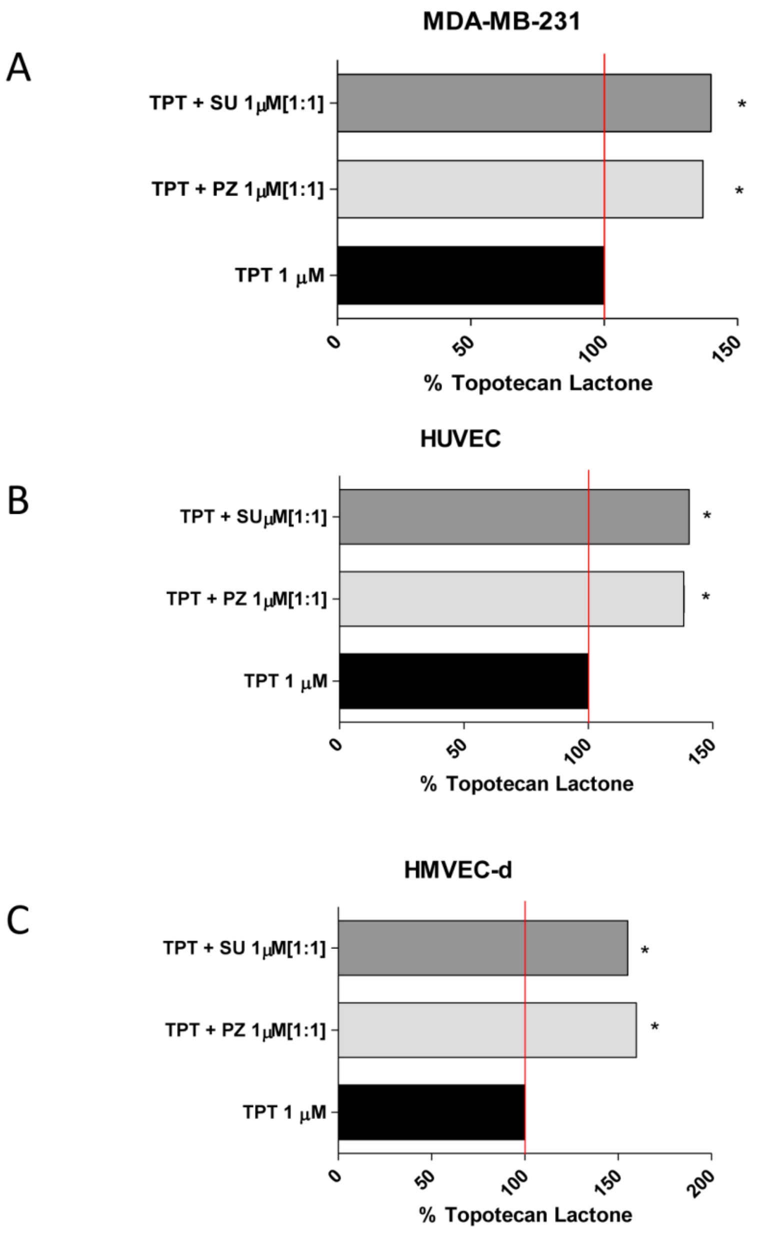

Supplementary figure 2. Accumulation of topotecan in MDA-MB-231 (**A**), HUVEC (**B**) and HMVEC-d (**C**) cell lines after exposure to 1 μM topotecan (TPT) alone and in combination with pazopanib (PZ) or sunitinib (SU). Columns and bars indicate mean values ± S.D., respectively. **P*<0.001 *versus* TPT. Small S.D. bars are not visible in the graph.

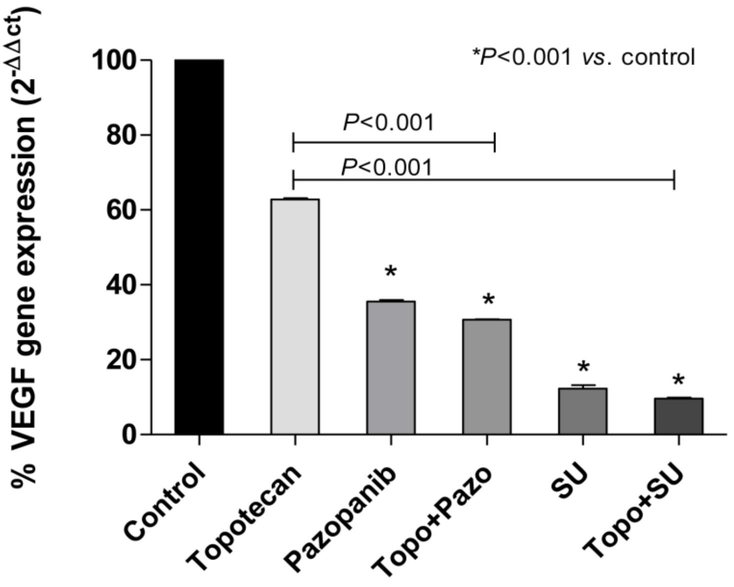

Supplementary figure 3. *VEGF* gene expression in 231/LM2-4 cells exposed to topotecan (Topo) , pazopanib (pazo), sunitinib (SU) and their combinations or with vehicle alone for 144 h in hypoxic conditions. Columns and bars, mean values ± S.E., respectively. Small error bars are not visible in the graph.

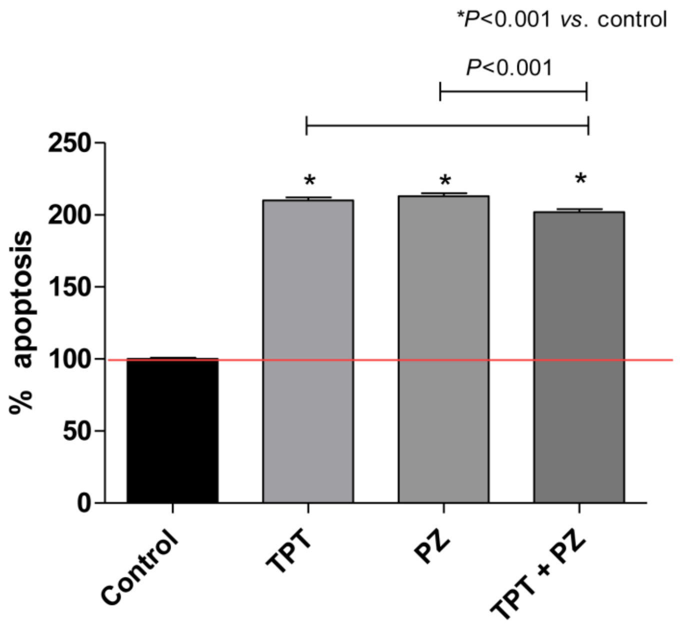

Supplementary figure 4. Proapoptotic effects of topotecan (TPT) and pazopanib (PZ) on HUVEC endothelial cells treated for 144 h. Apoptosis measurements using the Cell Death Detection ELISA Plus kit (Roche Diagnostics). % Absorbance values are representative of HUVEC cytosolic nucleosomes. Columns and bars, mean values ± S.E., respectively.
